# Epidemiological study on dengue in southern Brazil under the perspective of climate and poverty

**DOI:** 10.1038/s41598-020-58542-1

**Published:** 2020-02-07

**Authors:** Lorena Bavia, Francine Nesello Melanda, Thais Bonato de Arruda, Ana Luiza Pamplona Mosimann, Guilherme Ferreira Silveira, Mateus Nóbrega Aoki, Diogo Kuczera, Maria Lo Sarzi, Wilson Liuti Costa Junior, Ivete Conchon-Costa, Wander Rogério Pavanelli, Claudia Nunes Duarte dos Santos, Rafael Carvalho Barreto, Juliano Bordignon

**Affiliations:** 1 Setor de Ciências da Saúde, Hospital de Clínicas, UFPR, Curitiba, 80060-900 Brazil; 2 Laboratório de Parasitologia Experimental, Departamento de Ciências Patológicas, UEL, Londrina, 86057-970 Brazil; 3 Laboratório de Virologia Molecular do Instituto Carlos Chagas, ICC/Fiocruz/PR, Curitiba, 81350-010 Brazil; 4 Laboratório de Ciências e Tecnologias Aplicadas em Saúde do Instituto Carlos Chagas, ICC/Fiocruz/PR, Curitiba, 81350-010 Brazil; 5 Secretaria Municipal de Saúde de Cambé, Cambé, 86181-300 Brazil; 6 Centro de Computação Científica e Tecnológica, CCCT/UTFPR, Curitiba, 80230-901 Brazil

**Keywords:** Viral infection, Epidemiology, Risk factors

## Abstract

Social and epidemiological aspects of dengue were evaluated in an important metropolitan area in southern Brazil, from August 2012 to September 2014. Demographic, clinical, serological data were collected from patients with acute dengue symptoms treated at public health system units (HSUs). A systematic approach to analyze the spatial and temporal distribution of cases was developed, considering the temporal cross-correlation between dengue and weather, and the spatial correlation between dengue and income over the city’s census tracts. From the 878 patients with suggestive symptoms, 249 were diagnosed as positive dengue infection (28%). Considering the most statistically significant census tracts, a negative correlation was found between mean income and dengue (*r* = −0.65; *p* = 0.02; 95% CI: −0.03 to −0.91). The occurrence of dengue followed a seasonal distribution, and it was found to be three and four months delayed in relation to precipitation and temperature, respectively. Unexpectedly, the occurrence of symptomatic patients without dengue infection followed the same seasonal distribution, however its spatial distribution did not correlate with income. Through this methodology, we have found evidence that suggests a relation between dengue and poverty, which enriches the debate in the literature and sheds light on an extremely relevant socioeconomic and public health issue.

## Introduction

Dengue is an arthropod-borne viral disease caused by four different virus serotypes (DENV 1, 2, 3 and 4), which are transmitted by *Aedes spp*. mosquitoes^1^. The increase in frequency and magnitude of dengue outbreaks represents a public health challenge. The disease is endemic in more than 100 countries in tropical and subtropical regions, and 128 countries are at risk of a dengue outbreak^2^. The global trend of dengue epidemiology is characterized by a rapidly expanding geographic distribution of the vector despite the ongoing control efforts^3–5^. The vector competence is highly sensitive to climate^6^, and so the climate change in recent decades may help explain the current expansion of the disease worldwide^7,8^. Dengue hyperendemicity, characterized by the circulation of more than one serotype, is reported in almost every country in South America, where epidemics occur cyclically every three to five years, with increasing frequency and size^2,4^. Although the first dengue vaccine has been approved in some countries, there are serious concerns regarding its safety and efficacy^9^. In addition, no anti-dengue drug has been approved for use in humans to date^10^. Thus, the vector control through surveillance and prognostic approaches is still essential to control epidemics, as well as reduce morbidity and mortality associated with dengue infections^4,11^.

The first report of a dengue-like illness epidemic in Brazil dates back to 1845 in the state of Rio de Janeiro (Southeast Region)^12^. Nowadays, Brazil accounts for more than half of the dengue cases in the Americas^13^. In 2013, the number of dengue cases in Brazil were estimated in more than 1.4 million, with an economic impact of approximately USD 300 million (about BRL 600 million at the time)^14,15^. The Southeast Region has the highest population among Brazil’s five Regions, and also consistently shows the highest number of dengue cases^15–17^. Contrarily, the incidence rates in southern Brazil are consistently low when compared to other regions, especially the Central-West Region, which presents the highest incidence of dengue infection per capita^15–17^. The South Region is formed by three states: Paraná, Santa Catarina and Rio Grande do Sul, and it represents 14% of the country’s population^18^. In 2013, almost 5% (approx. 67,000) of the 1.4 million of dengue cases occurred in the southern Brazil^15^. From these 67,000, about 99% (approx. 66,000) occurred in Paraná, resulting in an incidence of approx. 600 cases/100,000 inhabitants^15^. In the same year, according to the State Health Department (*Secretaria de Estado da Saúde*, SESA) of Paraná, more than half of the municipalities had confirmed dengue cases, and about one third of all cities of Paraná had epidemic outbreaks (characterized by more than 300 cases per 100,000 inhabitants in the period considered)^19,20^. The majority of these cities are concentrated along the western, northwestern and northern Paraná state, close to the border with São Paulo state (Southeast Region), Mato Grosso do Sul state (Central-West Region), and Paraguay (endemic neighbouring country)^21^. These areas present significant cross-border commuting to work and study, which has a direct impact on the spread of epidemics^22–26^.

Taking into consideration the importance of dengue for public health, there are relatively few epidemiological studies on dengue in Brazil, especially in the southern region^27–29^. In an attempt to help filling this gap, social and epidemiological aspects of dengue notifications were investigated in the city of Cambé, located in an endemic metropolitan region in the southern Brazil. Cambé has about 100,000 inhabitants with a population density of 212 inhabitants/km^2^ and a degree of urbanization of 96%. It is bordered to the east by Londrina, the second largest city in the state of Paraná, located in the core of a metropolitan area with a population of one million inhabitants and a GDP of nine billion USD^30^. The aim of this study was to evaluate the demographic and serological characteristics of dengue patients in Cambé over a period of two years, to investigate the temporal cross-correlation of dengue occurrence with weather, and to analyze its correlation with poverty over the city’s census tracts.

## Results

### Dengue virus patients

**Figure 1:**
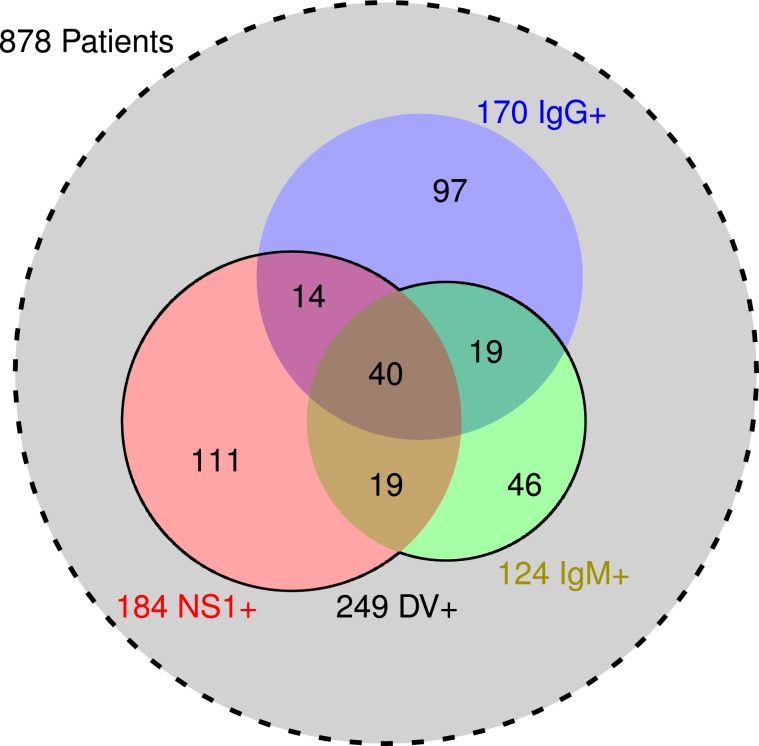
Serological distribution of the patients. The sets of serum samples positive for nonstructural protein 1 (NS1+, red), immunoglobulin M (IgM+, green) and G (IgG+, blue) are represented by circles whose areas are proportional to the number of patients. The values at the intersections are presented. The solid line represents the set of serum samples positive for dengue (DV+), which is the union of NS1+ and IgM+. The dashed circle in light grey represents all 878 patients.

Between August 2012 to September 2014, 878 patients fulfilled the requisites and accepted to take part in the study. The demographics and serological characteristics of these patients are shown in Table 1, while Fig. 1 presents a Venn diagram with the serological distribution of the patients. From the 878 patients, 249 (28%) were positive for dengue virus (DV+) and 629 samples (72%) negative (DV−). In the studied population, 170 samples (19% of 878) were immunoglobulin G positive (IgG+), of which 166 (98% of 170) were collected up to 4 days after the first reported symptoms, characterizing secondary infection. Considering the studied population, the incidence of primary dengue infection (DV+/IgG−) was higher when compared to secondary dengue infection (DV+/IgG+). However, dengue incidence was higher for IgG+ patients (43% of 170), when compared to IgG− (25% of 708), with a risk ratio of 1.7 (95% CI: 1.4 to 2.1). Among the 249 DV+ samples, 184 (74%) were positive for nonstructural protein 1 (NS1+) and 124 (50%) were positive for immunoglobulin M (IgM+), of which only 59 (24%) were simultaneously NS1+ and IgM+ (Fig. 1). NS1+ and IgM+ were negatively correlated (*r* = −0.60, 95% CI: −0.51 to −0.67), IgM+ and IgG+ were positively correlated (*r* = 0.40, 95% CI: 0.29 to 0.50), and no correlation was found between NS1+ and IgG+.

**Table 1:** Demographic and serological characteristics of patients with acute dengue symptoms from the city of Cambé, Paraná, Brazil, 2012-2014.

Parameters			DV+	DV−	*p* value
Patients		878 (100%)	249 (28%)	629 (72%)	—
Serology	NS1+ NS1−	184 (21%) 694 (79%)	184 (100%) 65 (9%)	0 (0%) 629 (91%)	—
	IgM+ IgM−	124 (14%) 754 (86%)	124 (100%) 125 (17%)	0 (0%) 629 (83%)	—
	IgG+ IgG−	170 (19%) 708 (81%)	73 (43%) 176 (25%)	97 (57%) 532 (75%)	<10^−5^
Gender	Female Male	464 (53%) 414 (47%)	122 (26%) 127 (31%)	342 (74%) 287 (69%)	0.150
Ethnic/skin color	White Pardo Black Yellow Indigenous	489 (56%) 144 (16%) 49 (6%) 8 (1%) 2 (< 1%)	139 (28%) 34 (24%) 17 (35%) 1 (12%) 2 (100%)	350 (72%) 110 (76%) 32 (65%) 7 (88%) 0 (0%)	0.071
	Not informed	186 (21%)	56 (30%)	130 (70%)	—
Age (years)		28 ± 17	32 ± 18	26 ± 16	<10^−5^
Pressure	SBP (mmHg) DBP (mmHg) MBP (mmHg)	109 ± 17 72 ± 12 85 ± 13	109 ± 16 72 ± 12 84 ± 12	110 ± 17 73 ± 13 85 ± 13	0.704 0.229 0.359

DV+ and DV− (positive and negative for dengue virus); NS1 (non-structural protein 1); Ig (immunoglobulin);

SBP (systolic blood pressure); DBP (diastolic blood pressure) and MBP (mean blood pressure).

**Figure 2:**
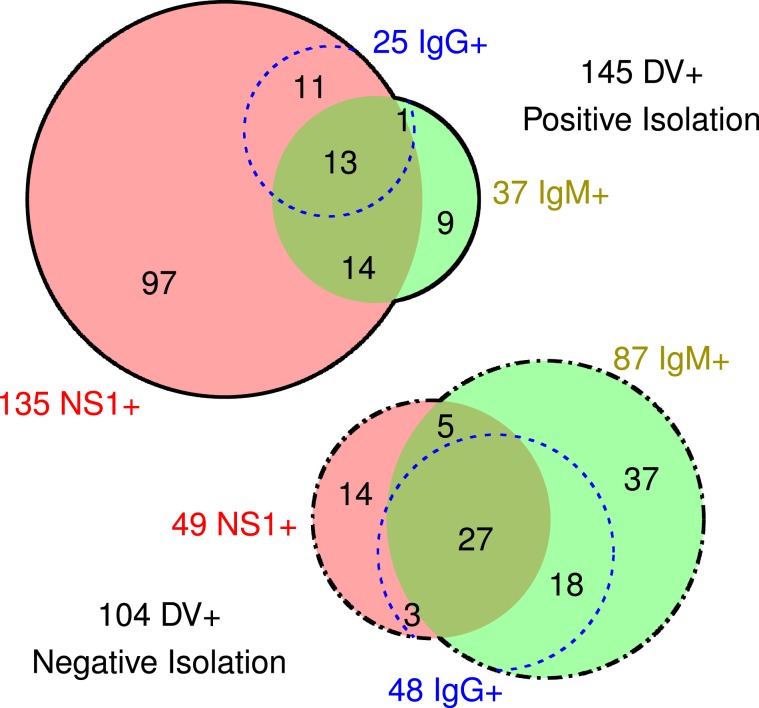
Dengue virus isolation of the 249 serum samples positive for dengue (DV+). The top diagram (solid line) represents the 145 successful virus isolation samples, while the bottom diagram (dash-dotted line) represents the 104 negative isolation. The sets of serum samples positive for nonstructural protein 1 (NS1+) and immunoglobulin M (IgM+) are represented respectively by circles in red and green, whose areas are proportional to the number of patients. The sets of serum samples positive for immunoglobulin G (IgG+) are represented by blue dotted open circles.

Using C6/36 cells, the virus strain was successfully isolated in 145 of the 249 DV+ serum samples (Fig. 2). Among these 145 positive samples, 135 (93%) were NS1+, and 37 (26%) were IgM+. In contrast, among the 104 DV+ samples with negative viral isolation, 49 (47%) were NS1+, and 87 (84%) were IgM+. The isolation was positively correlated with NS1+ (*r* = 0.52, 95% CI: 0.42 to 0.60), but negatively correlated with IgM+ (*r* = −0.58, 95% CI: −0.49 to −0.66) and IgG+ (*r* = −0.32, 95% CI: −0.21 to −0.43). Using a one-step RT-PCR assay, dengue virus serotype was identified in 140 samples from the 145 successful isolations. From these 140 samples, 139 were identified as DENV-1 and only 1 sample was DENV-4.

The mean age of the DV+ and DV− patients were respectively 32 ± 18 years and 26 ± 16 years (Table 1). There was no statistical difference considering gender. Among the 249 DV+ patients, 49% were male and 51% were female. Regarding ethnicity, the majority of the patients (56% of 878) declared themselves as white, followed by pardo (16%), black (6%), yellow and indigenous (less than 1% each), and no information was provided for 21% of the patients. Among the 249 DV+ patients, a similar ethnic distribution was found. No difference was found in systolic, diastolic and mean blood pressure in both DV+ and DV− patients (Table 1).

**Figure 3:**
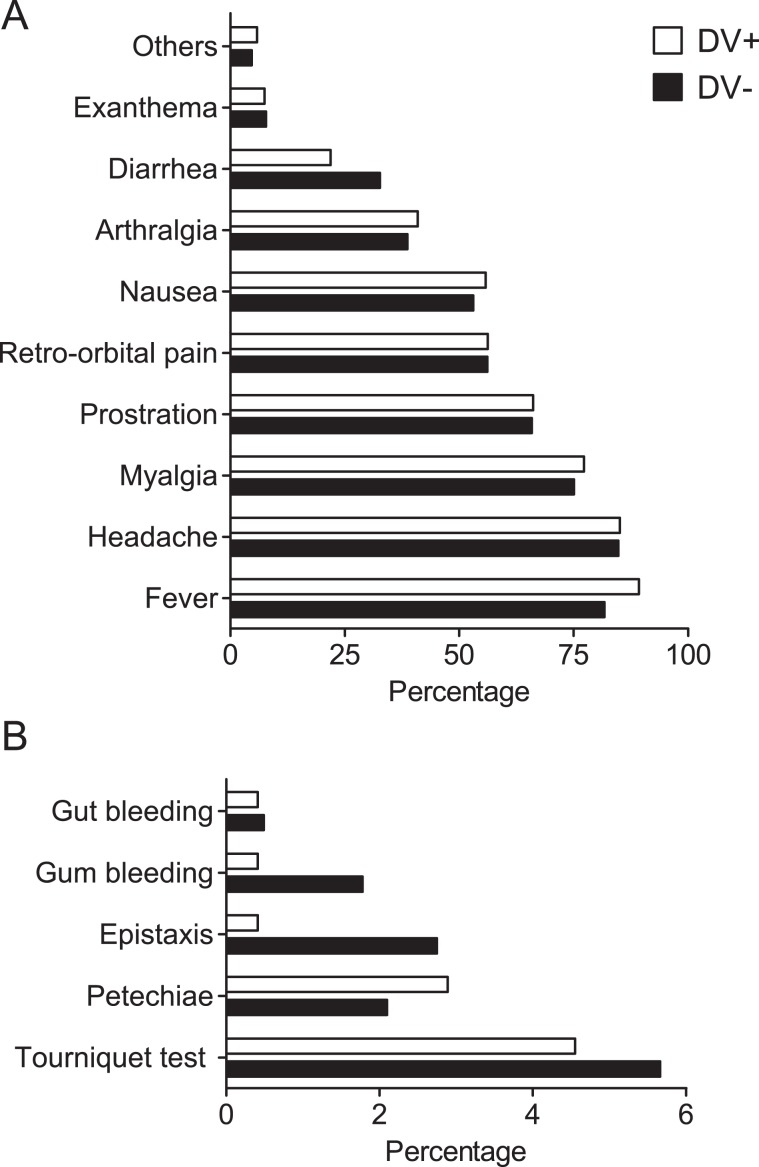
Symptomatology. Clinical (**A**) and hemorrhagic (**B**) manifestations in patients positive for dengue infection (DV+) compared to patients negative for dengue infection (DV−) included in the study.

Regarding the clinical aspects, the most frequent symptom observed in the 249 DV+ patients was fever (87%), followed by headache (83%), myalgia (75%), prostration (64%), retro-orbital pain (55%), nausea (54%), arthralgia (40%), diarrhea (21%), and exanthema (7%) (Fig. 3A). Other symptoms such as abdominal pain, backache, lethargy, pallor, dizziness, pruritus, and chills were reported by 8% of the DV+ patients. Similarly, the most frequent symptoms in the 629 DV− patients were headache (83%), fever (80%), myalgia (74%), prostration (65%), retro-orbital pain (55%), nausea (52%), arthralgia (38%), and diarrhea (32%).

Hemorrhagic manifestations were found in 80 patients (9% of 878 patients), and only 15 of them were DV+ (6% of 249). The most frequent hemorrhagic manifestation in DV+ patients was the presence of a positive tourniquet test (4%), followed by petechiae (3%). Epistaxis, gum bleeding and gut bleeding manifested in only one patient each (less than 1%). For the 629 DV− patients, 10% presented at least one hemorrhagic manifestation. The tourniquet test was the most frequent hemorrhagic manifestation, present in 6% of the DV− patients, followed by epistaxis (3%), petechiae (2%), gum bleeding (2%) and gut bleeding (less than 1%) (Fig. 3B). Metrorrhagia and hematuria were observed in 4 DV− patients each (less than 1%). Among the 249 DV+ patients, no correlation was found between any hemorrhagic symptoms and IgG+. As well, among all patients, no correlation was found between any clinical and serological aspects.

### Geographic characteristics and spatial distribution of dengue cases

**Figure 4:**
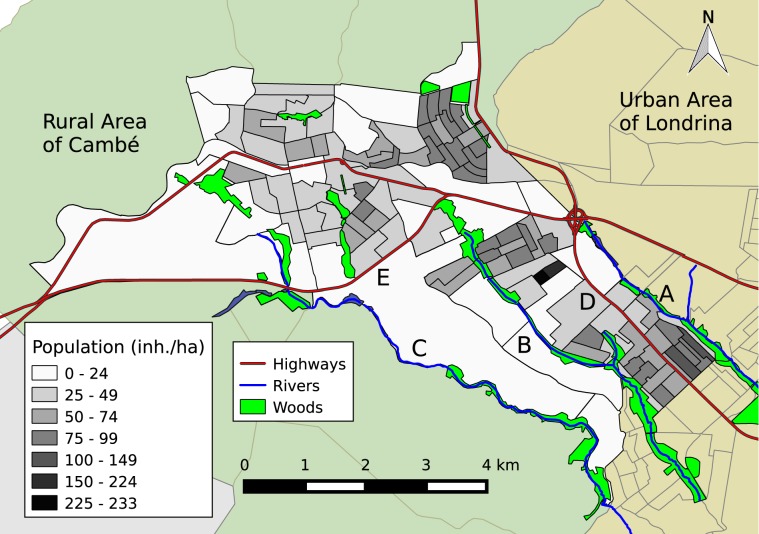
Geographic characteristics of the city of Cambé. The census regions and the population density of the urban area of Cambé, including the surrounding highways, bodies of water and woods. Cambé river (**A**); Esperança river (**B**); Cafezal river (**C**); Celso Garcia Cid highway – PR-445 (**D**); Melo Peixoto highway – BR-369 (**E**).

**Figure 5:**
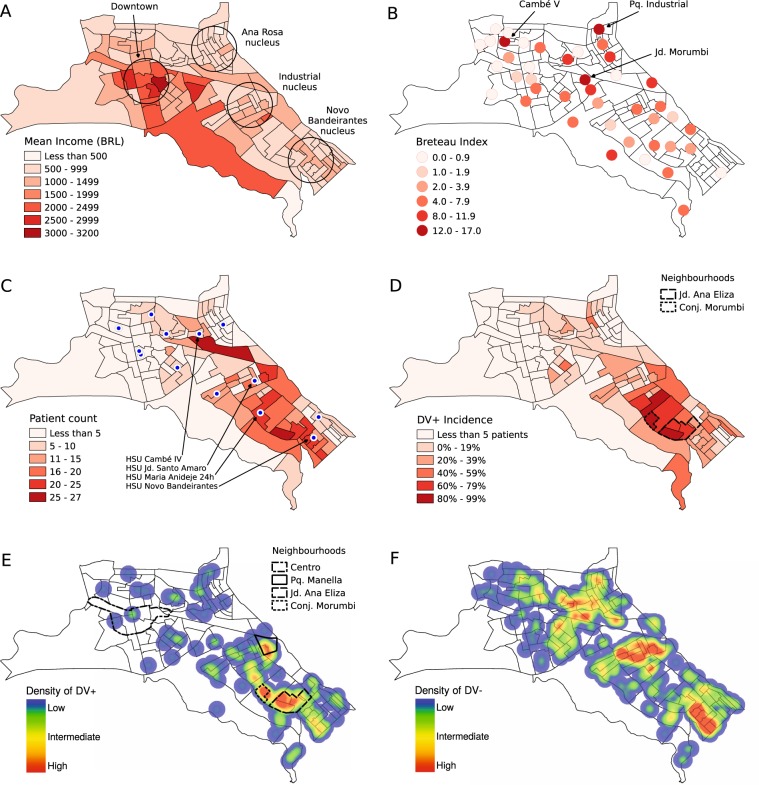
Geographic and epidemiological characteristics of the city of Cambé. The mean income distribution over the census tracts, showing the urban nuclei: downtown Cambé, *Ana Rosa*, *Industrial* and *Novo Bandeirantes* (**A**). The mean Breteau Index evaluated at February 2014 and April 2014, just before the 2014 outbreak (**B**). The HSUs where the patients were treated are presented as blue circles, as well as the number of patients treated over the census tracts (**C**). The accumulated data, from August 2012 to September 2014, regarding the dengue incidence considering only the census tracts with 5 or more patients (**D**). The spatial density of dengue patients (DV+) as a heatmap, highlighting the peripheral neighbourhoods with highest incidence: *Parque Residencial Manella*, *Jardim Ana Eliza* (II and III), and *Conjunto Habitacional Morumbi* (**E**). The main neighbourhood in downtown Cambé (*Centro*) is presented for comparison. The spatial density of patients negative for dengue infection (DV−) as a heatmap (**F**).

Physical and human characteristics of the urban area of Cambé are depicted in Fig. 4. It presents three small rivers that flow to the southeast and two highways that crosses the city. Cambé presents four main urban nuclei, including downtown and three peripheral regions (Fig. 5A). *Ana Rosa* nucleus is located farther north. *Industrial* nucleus is located next to the cloverleaf interchange. *Novo Bandeirantes* nucleus is located next to the southeast edge of the city. Compared to downtown, these three peripheral regions present a higher population density and a lower mean income. For the sake of the analysis, in relation to *Melo Peixoto* highway, *Ana Rosa* and downtown correspond to the “northern nuclei”, while *Industrial* and *Novo Bandeirantes* correspond to the “southern nuclei”. According to IBGE^31^, the northern nuclei had a total population of 51 thousand inhabitants, with a mean income of BRL 1230, while the southern nuclei had a total population of 41 thousand inhabitants, with a mean income of BRL 990.

The Breteau Index (BI) was intermittently evaluated seven times from August 2012 to July 2014. However, with the exception of February 2014 and April 2014 the results were not significant, that is, the BI was negligible. Figure 5B shows the Breteau Index over the urban area considering the mean value of February and April 2014, immediately before the 2014 outbreak. The location of the inspection spots are approximate, since the SMS provides only the name of the neighbourhoods, which mismatch the division of the census tracts. The BI was evaluated in 42 neighbourhoods, with 25 of them located in the northern nuclei and 17 in the southern nuclei. There were 20 neighbourhoods with *BI* > 4. The neighbourhood with the highest BI was *Parque Industrial* (*BI* = 16.6), followed by *Cambé V* (*BI* = 14.8), and *Jardim Morumbi* (*BI* = 14.7). These three neighbourhoods are located in the northern nuclei (Fig. 5B). The mean BI over the northern and southern nuclei were 4.6 ± 1.0 and 3.8 ± 0.6, respectively. In order to evaluate the correlation between BI and other spatial distributions, the BI value was considered for the neighbourhood’s nearest census tract. However, no significant correlation was found between the BI and any demographic aspect.

Figure 5C shows the number of patients on each census tract, and the locations of the public HSUs. The HSUs that took care of most patients were located in the southern nuclei: HSU *Jd. Santo Amaro*, HSU *Novo Bandeirantes*, and HSU *Maria Anideje 24 horas*, with 183, 174 and 166 patients respectively. The highest relative number of DV+ cases were found in HSU *Maria Anideje 24 horas* with 64%, followed by HSU *Jd. Santo Amaro* and HSU *Novo Bandeirantes*, both with 26%. These three HSUs treated 199 DV+ patients (80% of 249) considered in this work. In the northern nuclei, the HSU that took care of most patients were HSU *Cambé IV*, with 117 treated patients. However, only 7 of them (6% of 117) were DV+.

Figure 5D shows the distribution of dengue incidence over the census tracts of Cambé from August 2012 to September 2014, considering only the census tracts with 5 or more patients. No spatial correlation was found between dengue and the Breteau Index. Over the 20 census tracts with *BI* > 4, only 9 of them (45%) had reported dengue cases. *Jardim Ana Eliza* presented the census tracts with the highest incidence (up to 87%), followed by *Conjunto Habitacional Morumbi* (up to 78%).

Figure 5E presents the density of accumulated dengue cases as a heat map, in which high density is approx. 2 cases per hectare. Although the disease was distributed throughout the urban area of Cambé, there was a higher concentration in *Industrial* and *Novo Bandeirantes* nuclei. The figure shows a path of high density cases following *Esperança river* and connecting both nuclei. Considering the number of dengue cases per 1000 inhabitants (‰), the census tracts with the highest values were located at *Jardim Ana Eliza* II and III (up to 20.5‰), followed by *Conjunto Habitacional Morumbi* (up to 15.4‰) and *Parque Manella* (up to 13.7‰). The southeast border of *Jardim Ana Eliza* III is an area of environmental degradation, situated in a valley bottom, which is subject to flood^32^. In this area, the mean income was BRL 780, which was among the lowest 22% of the city. The spatial density of accumulated DV− cases as a heat map is presented in Fig. 5F for comparison. It is noteworthy that there were patients with the same symptoms of dengue over all the city.

**Figure 6:**
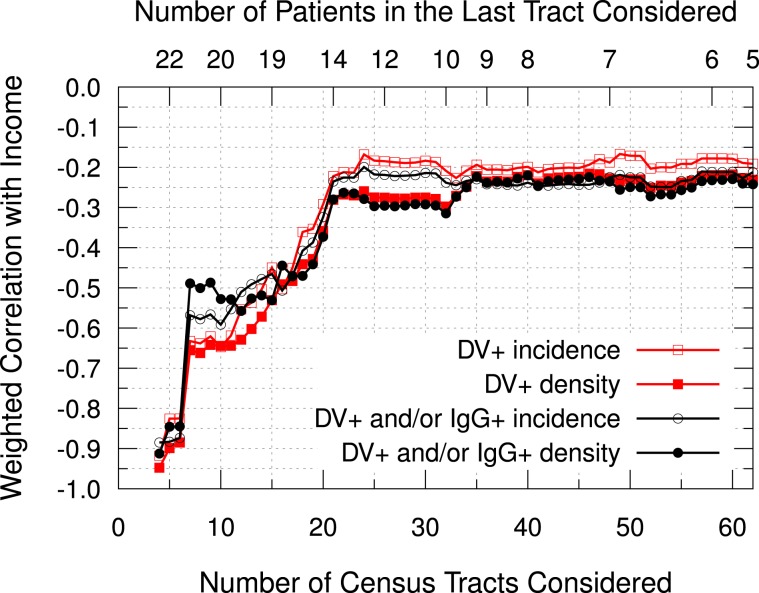
Accumulated weighted Pearson Correlation between dengue and income over the census tracts. Red squares represent dengue patients (DV+), while black circles represent the “Not Naïve for Dengue” patients (DV+ and/or IgG+, NND). Open geometric figures represent the correlation calculated with the incidence (cases per total patients), while the solid ones represent the correlation calculated with the density of cases (cases per inhabitants). The census tracts were ordered from the highest to the lowest number of patients. The top axis presents the number of patients in the last tract considered.

Figure 6 presents the accumulated weighted correlation between dengue and income over the census tracts, considering 849 patients with residence in the urban area of Cambé. From the 878 patients considered in the serology section, 20 were living in other cities and 9 were living in the rural area. From the 113 census tracts of the city, 103 presented at least one patient, and 62 presented 5 or more. Considering the density of dengue cases (DV+ patients per inhabitants) over the 103 census tracts with at least one patient, there was a weak negative correlation with the income (*r* = −0.24, 95% CI: −0.05 to −0.41). If the IgG+ patients are included along with the DV+ patients (“Not Naïve for Dengue” or NND), the negative correlation increases (*r* = −0.27, 95% CI: −0.08 to −0.44). When considering incidence, the correlation loses significance for both DV+ and NND groups. However, if the analysis is restricted to the census tracts with more patients (Fig. 5C), a moderate or even strong negative correlation is observed. Considering only the census tracts with 20 or more patients (total of 10 areas), a moderate negative correlation between DV+ incidence and income is found (*r* = −0.65, 95% CI: −0.03 to −0.91). The outbreak is located mostly over these tracts, in which 6 are located in *Novo Bandeirantes*, 3 are located in *Industrial*, and 1 is located in *Ana Rosa* (Fig. 5C–E). In order to understand if these results are a statistical artifact, the correlation between the density of patients (patients per inhabitants) and the mean income was analyzed over the census tracts. However, no significant correlation was found, no matter the number of census tracts considered.

**Figure 7:**
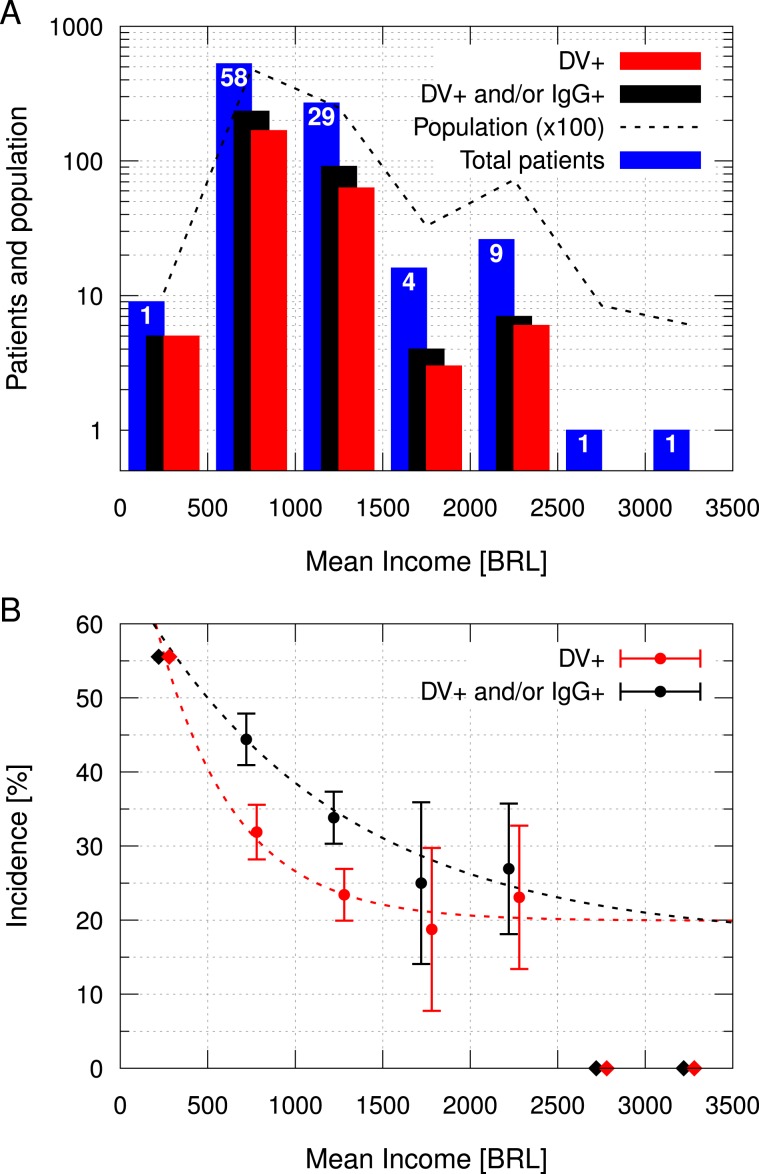
Distribution of patients and incidence by income, grouped in BRL 500 intervals. Dengue patients (DV+), patients “Not Naïve for Dengue” (DV+ and/or IgG+, NND), total number of patients and number of inhabitants (in hundreds) for each income interval (**A**). The blue columns represent the number of patients in each interval, while black and red columns represent the NND and DV+ patients, respectively. The dashed line represents the number of inhabitants (in hundreds), and the numbers in the top of the blue columns represent the number of census tracts grouped in each interval. Incidence (cases per total patients) distributed by income (**B**). The uncertainties refer to the statistical error, calculated using the weighted mean over the census tracts grouped each income interval. There was only one census tract with less than BRL 500, and only two with more than BRL 2500, which were represented by diamonds without uncertainty. Exponential decay fits are represented by dashed lines.

Figure 7A presents a histogram of patients, DV+ and NND grouped by income in seven BRL 500 intervals. Most patients (62%) were located in 58 census tracts with mean income in the BRL 500–1000 range. The second largest group (32%) was in BRL 1000–1500 range, with 29 census tracts. Therefore, most patients were located in census tracts with mean income below the city’s average, which was BRL 1120. This patients distribution was consonant with the inhabitants distribution in the census tracts considered, which is presented with a dashed line in Fig. 7A. Over these seven income intervals, the Pearson correlation coefficient between the patients number and inhabitants number was 0.996 (95% CI: 0.972 to 0.999). Complementarily, Fig. 7B presents the mean incidence of DV+ and NND by income using the same intervals of Fig. 7A. The uncertainties are the statistical error considering the weighted mean over the census tracts. Both DV+ and NND incidences were 56% in *Campos Verdes*, the only census tract with less than BRL 500, located in the southernmost part of the city. For this interval, the uncertainty was estimated in one case (about 11%), and it was not presented in the graph. From BRL 500–1000 range to BRL 1000–1500 range, the mean DV+ incidence reduced from 32% to 23%, and the mean NND reduced from 44% to 34%. Although incidences have reduced in the BRL 1500–2000 range, their uncertainties are relatively large. The intervals over BRL 2500 had one patient each and zero positive cases, thus they were not considered in the analysis because it was not possible to estimate their uncertainties. Considering the uncertainties, only the incidences between BRL 500 and BRL 1500 are relevant.

Table 2 presents the number of dengue cases (DV+) and previous infection (IgG+) among the urban nuclei of Cambé. The incidence of DV+ was higher in the southern nuclei (38% of 575) when compared to the northern nuclei (10% of 274), with a risk ratio of 3.7 (95% CI: 2.6 to 5.3). Downtown is the wealthiest nucleus, and presented a low incidence of DV+ (12% of 118) and low prevalence of IgG+ (14% of 118) when compared to the peripheral nuclei. *Novo Bandeirantes* has the lowest mean income, and presented the highest incidence of DV+ (44% of 338) and highest prevalence of IgG+ (25% of 338) among all nuclei. When comparing *Novo Bandeirantes* to downtown, the estimated risk ratio for DV+ was 3.7 (95% CI: 2.3 to 6.2), and the prevalence ratio for IgG+ was 1.8 (95% CI: 1.1 to 3.0). *Ana Rosa* has the second lowest mean income, presented the second highest prevalence of IgG+, but the lowest incidence of DV+. Over all nuclei, there was a higher incidence of dengue infection in IgG+ patients when compared to IgG−.

**Table 2:** Mean income (BRL), dengue infection (DV+) and previous contact with dengue (IgG+) over the urban nuclei.

Nuclei	BRL	Urban Patients	DV+	DV−	IgG	Total/IgG	DV+/IgG	DV−/IgG	*p* value*
No stratification	1120	849 (100%)	245 (29%)	604 (71%)	+ −	166 (20%) 683 (80%)	70 (42%) 175 (26%)	96 (58%) 508 (74%)	<10^−4^
Downtown Ana Rosa Industrial Novo Bandeirantes	1480	118 (14%)	14 (12%)	104 (88%)	+ −	16 (14%) 102 (86%)	5 (31%) 9 (9%)	11 (69%) 93 (91%)	<10^−18^
	870	156 (18%)	14 (9%)	142 (91%)	+ −	32 (21%) 124 (79%)	4 (13%) 10 (8%)	28 (88%) 114 (92%)	
	1180	237 (28%)	67 (28%)	170 (72%)	+ −	35 (15%) 202 (85%)	18 (51%) 49 (24%)	17 (49%) 153 (76%)	
	840	338 (40%)	150 (44%)	188 (56%)	+ −	83 (25%) 255 (75%)	43 (52%) 107 (42%)	40 (48%) 148 (58%)	
Northern Southern	1230	274 (32%)	28 (10%)	246 (90%)	+ −	48 (18%) 226 (82%)	9 (19%) 19 (8%)	39 (81%) 207 (92%)	<10^−17^
	990	575 (68%)	217 (38%)	358 (62%)	+ −	118 (21%) 457 (79%)	61 (52%) 156 (34%)	57 (48%) 301 (66%)	

*Note: The *p* value refers to the rightmost columns, that is, the DV/IgG distribution over the nuclei.

Considering the 103 census tracts with at least one patient, a barely strong positive correlation (*r* = 0.67, 95% CI: 0.54 to 0.76) was found between primary (DV+/IgG−) and secondary (DV+/IgG+) density of dengue cases over the census tracts.

### Seasonal distribution of dengue cases

**Figure 8:**
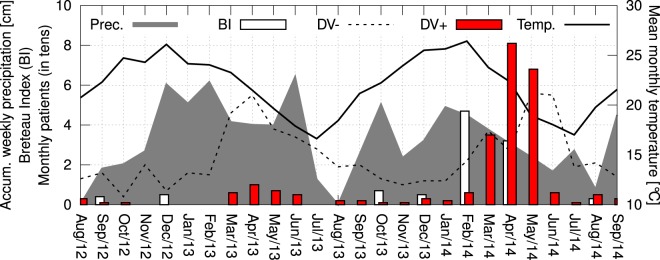
Seasonal distribution of dengue cases, temperature and precipitation in the city of Cambé. The red bars represent the monthly occurrence of patients positive for dengue (DV+), the dashed line represents the number of patients negative for dengue (DV−), the white bars represent the Breteau Index (BI), the shaded area represents the monthly average of the accumulated weekly precipitation (in cm), and the line indicates the mean monthly temperature (in °C), from August/2012 to September/2014.

The monthly DV+ occurrence from August 2012 to September 2014 is presented in Fig. 8. The incidence is seasonally distributed, with an increase in the number of DV+ patients from March to May in both years of the study. For comparison, Fig. 8 shows the monthly number of DV− patients, which is also seasonal. Using the Spearman’s rank correlation coefficient, and considering the monthly occurrence of DV+ and DV− patients, a barely strong correlation (*r* = 0.65, 95% CI: 0.36 to 0.83) was found over the period of study. In 2014, from March to May, there were 184 DV+ patients, representing 74% of all 249 dengue cases. In these three months, there were 304 patients, which means that 61% of these patients were DV+. April was the month with the highest number of cases, with 10 cases in 2013 and 81 cases in 2014. Over the period of study, the Breteau Index was evaluated seven times. In February 2014, the BI reached 4.7. Figure 8 also shows that the monthly mean temperature reached 26.1 °C in December 2012 and 26.4 °C in February 2014. The monthly average of the accumulated weekly precipitation reached 6.43 cm in June 2013 and 4.90 cm in January 2014. From August 2012 to July 2013, the accumulated precipitation was 175 cm, while in the same period one year later the accumulated precipitation was 145 cm.

**Figure 9:**
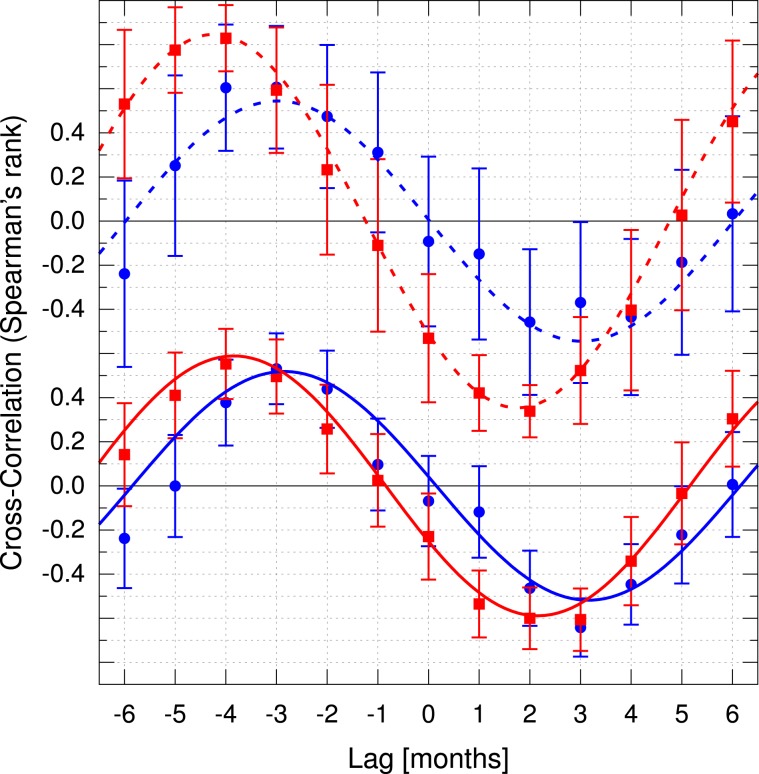
Cross-correlation of temperature (red squares) and precipitation (blue circles) with the number of symptomatic patients negative for dengue (dashed lines) and positive for dengue (solid lines). The error bars represent one standard deviation, and a single harmonic function was used to fit the curves to determine the delays.

In order to understand the effect of the weather on dengue occurrence, both temperature and precipitation were cross-correlated with the number of patients, considering a displacement of up to 6 months (Fig. 9). The time lag (in months) between precipitation and DV+ was *τ*_0_ = −2.8 ± 0.2 (*r* = 0.52 ± 0.04), and temperature and DV+ was *τ*_0_ = −3.8 ± 0.1 (*r* = 0.59 ± 0.02). Similarly, for precipitation and DV− was found *τ*_0_ = −3.0 ± 0.2 (*r* = 0.54 ± 0.04), and for temperature and DV−, *τ*_0_ = −4.24 ± 0.05 (*r* = 0.85 ± 0.01). The negative time lag values indicate that precipitation and temperature raised before the increase in the number of patients.

Table 3 presents the dengue occurrence among the urban nuclei in two time periods: “year 1” (until August 31st 2013) and “year 2” (after August 31st 2013). The DV+ incidence in year 2 (41% of 514) was higher when compared to year 1 (10% of 335), with a risk ratio of 3.9 (95% CI: 2.8 to 5.4). In year 1, the amount of patients was approximately 50% in north and south. From year 1 to year 2, the number of patients treated in the northern nuclei decreased by 43%, while there was a increase of 157% in the south. Despite the reduction in the number of patients treated in the northern nuclei, the number of DV+ almost doubled. In contrast, in the south, the number of patients as well as the number of DV+ increased significantly. In both regions, the incidence of DV+ in year 2 was higher than year 1, with risk ratios of 3.0 (95% CI: 2.1 to 4.4) for the southern nuclei, and 3.1 (95% CI: 1.5 to 6.5) for the north. Equivalently, in both periods, the incidence of DV+ in the southern nuclei was higher when compared to the north, with risk ratios of 2.7 (95% CI: 1.3 to 5.5) for year 1, and 2.6 (95% CI: 1.7 to 4.0) for year 2.

**Table 3:** Dengue infection over the urban nuclei by year.

Nuclei	Patients	DV+	DV−
Year 1	335 (100%)	35 (10%)	300 (90%)
Northern	174 (52%)	10 (6%)	164 (94%)
Southern	161 (48%)	25 (16%)	136 (84%)
Year 2	514 (100%)	210 (41%)	304 (59%)
Northern	100 (19%)	18 (18%)	82 (82%)
Southern	414 (81%)	192 (46%)	222 (54%)

Note: *p* = 0.005 for year 1, and *p* < 10^−5^ for year 2.

### Correlations and risk ratios

Table 4 summarizes the three groups of correlations calculated in this work, regarding the serology and clinical symptoms over the 249 DV+ patients, the spatial distributions of dengue incidence, population density and income over the 103 census tracts with at least one patient, and the seasonal distributions of dengue incidence, temperature and rainfall over the 26 months from August 2012 to September 2014. Table 5 summarizes the dengue risk ratios evaluated in this work, considering the previous contact (presence of IgG), the comparison between southern and northern urban nuclei, the poorest and the wealthiest nucleus, and year 1 and 2 (with and without outbreak).

**Table 4:** Summary of evaluated correlations in the order they are discussed in this work.

Variables	Sample Size	Correlation	95% CI
**Serology and Clinical symptoms (Correlation model: Pearson)**			
NS1/IgM	249 patients	−0.60	−0.51 to −0.67
IgM/IgG	249 patients	0.40	0.29 to 0.50
NS1/IgG	249 patients	Not significant	
Virus Isolation/NS1	249 patients	0.52	0.42 to 0.60
Virus Isolation/IgM	249 patients	−0.58	−0.49 to −0.66
Virus Isolation/IgG Hemorrhagic	249 patients	−0.32	−0.21 to −0.43
Manifestations/IgG	249 patients	Not significant	
Symptoms/Serology	249 patients	Not significant	
**Spatial distributions (Correlation model: Weighted Pearson)**			
BI/Demographic ^a^	42 census tracts	Not significant	
BI/Dengue Incidence ^b^	42 census tracts	Not significant	
Dengue Density ^c^/ Mean Income	103 census tracts	−0.24	−0.05 to −0.41
NND Density ^d^/ Mean Income	103 census tracts	−0.27	−0.08 to −0.44
Dengue Incidence/ Mean Income	10 census tracts	−0.65	−0.03 to −0.91
Patients Density ^e^/ Mean Income	10–103 census tracts	Not significant	
Primary/Secondary Dengue Incidence	103 census tracts	0.67	0.54 to 0.76
**Seasonal distribution (Correlation model: Spearman)**			
DV+/DV− Monthly Occurrence	26 months	0.65	0.36 to 0.83
Precipitation/DV+ Monthly Occurrence	20–26 months*	0.52 ± 0.04^†^	^†^
Temperature/DV+ Monthly Occurrence	20–26 months *	0.59 ± 0.02^†^	†
Precipitation/DV− Monthly Occurrence	20–26 months *	0.54 ± 0.04^†^	^†^
Temperature/DV− Monthly Occurrence	20–26 months *	0.85 ± 0.01^†^	^†^

^a^Population per hectare or mean income in the census tracts.

^b^DV+ patients per number of patients (DV+ and DV−) in the census tracts.

^c^DV+ patients per population in the census tracts.

^d^DV+ and/or IgG+ patients per population in the census tracts.

^e^Number of patients (DV+ and DV−) per population in the census tracts.

*Cross-correlation scanning from −6 months to +6 months.

^†^This is the result of a fitting. The 95% confidence interval was not evaluated.

**Table 5:** Summary of evaluated dengue risk ratios in the order they are discussed in this work.

Dengue Risk	Sample Size	Ratio	95% CI
IgG+ over IgG−	878 patients	1.7	1.4 to 2.1
Southern over northern nuclei	849 patients	3.7	2.6 to 5.3
Poorest over wealthiest nucleus	456 patients	3.7	2.3 to 6.2
Southern over northern nuclei^a^	335 patients	2.7	1.3 to 5.5
Southern over northern nuclei^b^	514 patients	2.6	1.7 to 4.0

^a^Year 1: From August 2012 to August 2013.

^b^Year 2: From September 2013 to September 2014 (Outbreak year).

## Discussion

It is possible to suggest an association between poverty and dengue in Cambé, from August 2012 to September 2014, by correlating dengue incidence and mean income over the census tracts. Throughout the period, the risk of dengue infection was higher in the poorer areas. The base rate bias have been avoided by carefully analyzing the spatial distribution of dengue cases considering the number of patients and the population in the respective census tracts.

Primary and secondary dengue cases were spatially correlated. This suggests that dengue is endemic only in certain areas of the city. These cases were concentrated along a bottom valley, where the mean income was 37% lower than the wealthiest urban nuclei. Despite the significant number of articles addressing dengue and poverty, the debate persists in the literature, and there is still no established relationship^33–35^. The main poverty indicators related to dengue include education, socioeconomic status, income, physical housing conditions and water supply^34^. Previous works have showed that the dengue risk is higher among families earning less than one minimum wage^36^, those living in poor household conditions which promote contact between hosts and vectors^37^, as well as those living in rural areas lacking piped water supply^33^. Few studies have shown a negative correlation between dengue cases and social factors, such as education and household conditions^35,38^. A moderate negative correlation was observed between the mean income and dengue (*r* = −0.65; *p* = 0.02) when considering the most statistically significant census tracts. The inclusion of census tracts with few patients, especially outside the outbreak area, may explain the weakening of the correlation. The intrinsic uncertainties and fluctuations characteristic of small number statistics, as well as several uncontrolled variables, could make the outbreak spatially heterogeneous, resulting in the reduction of any possible correlation with socioeconomic aspects. This could explain why most of the previous works did not find spatial correlation between dengue and poverty indicators. However, by grouping the patients in mean income intervals, it was possible to observe a negative trend between dengue and income. The mean dengue incidence reduced from 32% to 23%, when the mean income raised from BRL 500–1000 to BRL 1000–1500. Likewise, the dengue risk ratio between the southern and the northern nuclei was 2.6–2.7 in both years, that is, with and without outbreak.

The risk ratio of 1.7 of dengue among IgG+ compared to IgG− supports that a previous contact to a different serotype could make the patients more susceptible to a following infection^39–41^. However, no significant relation was found between the presence of IgG+ and hemorrhagic manifestations among the DV+ patients. Such phenomena was observed by Watts *et al*.^42^, in which DENV-2 did not cause dengue hemorrhagic fever nor dengue shock syndrome in patients previously infected with DENV-1 in Peru in 1995. However, studying the same population, Kochel *et al*. showed that sera positive for the DENV-1 antibody where capable to neutralize the circulating DENV-2^43^. Based on this result, Kochel *et al*. suggested the absence of dengue hemorrhagic fever in individuals infected with DENV-1 followed by DENV-2 in Peru in 1995 could be explained by cross-neutralization. If this is the case in the patients from Cambé from 2012 to 2014, the higher incidence of dengue among the IgG+ could be explained by other factors, such as a sampling bias produced by an inhomogeneous spatial distribution of the endemic. In other words, over the years, a significant high infection rate in some areas could increase DV+/IgG+ over DV+/IgG−, while a systematic low infection rate in other areas could decrease DV−/IgG+ over DV−/IgG−.

From the 878 patients with suggestive dengue symptoms, only 249 (28%) were found to be positive for dengue virus. This is not unusual and may be due to the similarity of dengue’s symptoms with other febrile diseases^44^. The most frequent symptoms in DV+ patients were: fever, headache, myalgia and prostration. Interestingly, 13% of the DV+ patients had no fever. Although it is classified as a febrile disease, the absence of this symptom in dengue infections is not rare, as shown in other clinic-epidemiological studies performed in Brazil^45^. It is estimated that approximately 3/4 of total dengue cases are asymptomatic infections^11^. Additionally, when considering the same population, distinct serotypes may produce different sets of symptoms^46^.

The dengue virus isolation in cell culture is related to the presence of NS1 and specific IgM/IgG dengue antibodies in the samples^47,48^. Here, we confirm that virus isolation in C6/36 was most effective on samples also positive for NS1 and without specific anti-dengue antibodies (IgM/IgG). Ahmed and Bhoor demonstrated a concordance of 89% between dengue NS1 antigen detection and virus isolation^49^. However, the presence of specific anti-dengue antibodies impairs virus isolation in C6/36 cells probably due to virus neutralization^49,50^. The virus strain was successfully isolated in 58% of the DV+ samples, and the serotypes were identified in 97% of these, in which 139 were DENV-1 and one was DENV-4. Although the DENV-4 patient was diagnosed in Cambé, it was a case imported from a neighbouring city^51^. The serotypes found are in consonance with the Health Surveillance Department (*Secretaria de Vigilância em Saúde*, SVS) reports for the state of Paraná in 2014^15^. According to the SVS, 901 samples were tested for serotype, in which 51% were successfully confirmed, with 98.9% DENV-1 and only 1.1% DENV-4. During the first decade of the 21st century, the predominant serotype circulating in Brazil was DENV-3, with a high incidence of DENV-1 in the beginning of the decade, and an increase of DENV-2 after 2005^16^. From 2005 to 2009, the predominant serotype circulating in Paraná was DENV-3, while from 2010 to 2016, there was a high incidence of DENV-1, with a small incidence of DENV-2^17,52,53^.

Among the DV+ patients, the negative correlation between NS1+ and IgM+ (*r* = −0.60) was expected. Moi *et al*. observed *r* = −0.62 in Japanese travelers who were infected while visiting endemic countries^54^. This negative correlation happens because the detection rate of NS1 decreases over a week following the onset of the symptoms, while IgM increases at the same time^55,56^.

Between February and April 2014, immediately before the 2014 outbreak, the Breteau Index was greater than 4 in 20 of 42 neighbourhoods. According to Sanchez *et al*., *BI* > 4 is indicative of epidemic outbreak^57,58^. Although the Breteau Index had predicted the 2014 outbreak in Cambé, no spatial correlation between the BI and the dengue occurrence was found. This is an interesting result previously reported by Bowman *et al*.^59^. This could be explained not solely by the sampling protocols, as suggested by Bowman *et al*., but also by the work conditions of the endemic disease control agents^60,61^. Despite the awareness campaigns^62,63^, these agents confront several issues to inspect the residences^64^, including aggression^65–69^ and distrust of the residents due to the risk of robbery^70,71^.

Although the outbreak in Cambé occurred in 2014, the number of confirmed cases in the state of Paraná decreased by about 40% from 2013 to 2014^19,20^. Considering the predominant circulating serotype can completely change in less than five years, as reported by Teixeira *et al*.^16^, the late outbreak could be explained by a delay in the virus spreading dynamics. This dynamics depends on several factors^72^, such as the commute network^24–26^, population’s immunization status^42,43^, vector availability^37^, and especially on weather factors such as wind, humidity, precipitation and temperature^6,73^.

Temperature and the number of DV+ patients presented a positive cross-correlation with a lag interval of four months. This means it is expected that the dengue cases will increase approximately four months after the increase in the temperature. The peak in the monthly mean temperature was approximately 26 °C, which is inside the range of favorable temperature (from 21 °C to 29 °C) for the development of the vector of dengue *Aedes aegypti*^74^. Recently, it was shown that higher temperatures (from 28 °C to 32 °C) impact in both vector competence of *Aedes albopictus* for DENV-2 transmission and in shortening the length of the extrinsic incubation period^6^. Regarding precipitation and dengue, there is a positive cross-correlation with a lag interval of three months. Likewise, the calculated maximum correlation between rainfall and dengue cases in Sri Lanka from 2003 to 2012 and in Bangladesh from 2010 to 2014 presented a lag of two months^73,75^. A delay of two months between maximum values of the monthly rainfall and dengue cases was also observed in Pirapora and Mossoró, two cities located respectively in the Southeast and Northeast Regions of Brazil^76,77^. The three month delay between rainfall and dengue incidence observed in our study, which is about one month higher than those described above, may be explained by the relatively lower average annual temperature of Cambé. In addition, similar cross-correlations between temperature and precipitation with the number of DV− patients were also found. Thus, despite the known connection between change in the weather and infectious diseases^78^, this could be an indication of the circulation of other mosquito-borne infectious diseases in the region. And last but not least, a new simple approach to analyze the cross-correlation function using a single harmonic fitting has been presented.

Finally the results bring new epidemiological information regarding the influence of weather and socioeconomic aspects of dengue in southern Brazil.

### Limitations

Only patients treated in the public health system units were considered and therefore there is no data on private diagnostic clinics. Although the sampled patient distribution correlated with the population distribution regarding income, knowledge about private diagnostic clinics could provide important information about the wealthiest urban nucleus, which could weaken or strengthen the results. The asymptomatic patients, which have an important role on the transmission dynamics of vector-borne diseases^72^, were not evaluated. It was considered a period of only twenty six months. Despite our calculated cross-correlation coefficients being significant, a longer period could reduce or confirm their strength. Also, a longer time range could provide important information about the weather and the dengue infection in Cambé, which could be used to understand why there were more dengue cases in 2014 than 2013. Apart from statistics of small numbers, there are uncontrolled factors which may explain the reduction of correlation when considering census tracts with fewer patients, such as the location where patient and vector came into contact (not necessarily the patients’ addresses), population density and commute network^26^, as well as microclimate variations in the neighbourhoods^79^. Although the Breteau Index have successfully predicted the 2014 outbreak, the surveys were few and intermittent, and no temporal cross-correlation with BI could be performed. Correlation is not synonymous with dependence or causation. The present work suggests that income and dengue were related in Cambé from 2012 to 2014, but not how. To understand how they were related would require a more detailed study with more data and multivariate analysis, considering education, household conditions, the proximity of bodies of water, forests and dumps, as well as neighbourhood microclimate and commute networks.

## Methods

### Study location, ethical statements and consent

This is a epidemiological study of the dengue occurrence in the city of Cambé, North Paraná (23;16;33S, 51;16;40W), South Region of Brazil, from August 2012 to September 2014. Signed informed consent was obtained from all patients at the time of sample collection. All adult patients provided informed consent, and a parent or guardian provided informed consent on the child’s behalf. The protocol for the study of human patients was approved by the Committee of Research Ethics from Fiocruz 617/11 and CAAE: 0038.0.011.000–11/Fiocruz. All experiments were performed in accordance with relevant guidelines and regulations.

### Patients

Patients with clinical suspicion of dengue virus infection, according to the WHO criteria^80^, and treated in the public HSU in the city of Cambé were invited to participate in the study. Clinical and epidemiological data, including patient’s address, ethnicity, gender, age, pressure, and symptoms, were retrieved from the Information System for Notifiable Diseases forms (*Sistema de Informação de Agravos de Notificação*, SINAN). This form is used to compulsorily notify at least seven neglected tropical diseases: dengue, acute Chagas disease, leishmaniasis, malaria, schistosomiasis, leprosy, and tuberculosis^81^. Blood samples were obtained by venipuncture after written consent of the patient. Serum samples collected from the patients were tested for dengue infection using Panbio’s Dengue Early ELISA for the detection of non-structural protein 1 (NS1), and Panbio’s IgM and IgG ELISA assays. Samples positive for NS1 and/or IgM were considered as acute phase dengue (DV+), which were selected for virus isolation in C6/36 cells, while the serotyping was performed via one-step RT-PCR, following the protocol described by Kuczera *et al*.^51^. Samples with negative or indeterminate outcomes for NS1 and IgM were considered negative to dengue virus infection (DV−). The presence of anti-dengue specific IgG until 7 days after the onset of symptoms is an indicative of secondary infection^47,48,82^. Patients negative for IgG were considered “naïve for previous infection”, while those positive for IgG were considered “previously infected patients”. Among the DV+ patients, those negative for IgG were counted as “primary dengue infection” (DV+/IgG−), while those positive for IgG were counted as “secondary dengue infection” (DV+/IgG+). Since the number of IgG+ patients was relatively small, naïve and previously infected patients were considered separately to avoid base rate bias. Samples DV+ and/or IgG+ were categorized as “Not Naïve for Dengue” or NND.

### Geographic and epidemiological characteristics

The cartographic base, the geographic characteristics and socioeconomic data of Cambé, including mean income and population in each census tract, were obtained from the Brazilian Institute of Geography and Statistics (*Instituto Brasileiro de Geografia e Estatística*, IBGE)^31,83^, the official government bureau for geostatistical data in Brazil. The census tracts were the geographic units used to analyze mean income and population density. The vector data regarding highways, woods and permanent water flows were retrieved from OpenStreetMap, and confirmed using satellite pictures from Google Maps. The georeferencing of the patients was retrieved using BatchGeo software tool, and confirmed through Google Maps. In each census tract, the dengue incidence was evaluated through the ratio of patients positive for dengue (DV+) to the total number of symptomatic patients (DV+ and DV−). The Breteau Index (BI) is defined as the number of containers positive for *Aedes* larvae and/or pupae per 100 houses inspected^84^. The BI was provided by the Cambé City Health Department (*Secretaria Municipal de Saúde de Cambé*, SMS)^85^, which intermittently evaluated it 7 times in the period of the study: twice in 2012, twice in 2013, and thrice in 2014. The QGIS tool was used to evaluate the number of patients and BI for each census tract, and to render the spatial distributions. In order to visualize the spatial distributions of patients in the city, the kernel density estimation for intensity was employed^86^, using a kernel radius of 300 m. The kernel density estimation was used for qualitative analysis. Daily meteorological data for temperature and rainfall were obtained from the Meteorological Database for Teaching and Research (*Banco de Dados Meteorológicos para Ensino e Pesquisa*, BDMEP)^87^. From the daily values, the monthly mean temperature, and the weekly and monthly accumulated precipitation were calculated. The monthly distribution of dengue cases and the monthly meteorological data were analyzed and cross-correlated considering the city as a whole.

### Statistical analysis

The descriptive analysis was performed by means of absolute and relative frequencies, measures of central tendency and dispersion. Pearson Chi-Square test was used in all contingency tables regarding the bivariate analysis of categorical variables, such as serology, gender, ethnic/skin color and urban nuclei stratification. For continuous variables, Mann-Whitney U test was used to verify the differences between the means using the same level of significance. The correlations between categorical variables such as serology and symptoms were evaluated using Pearson correlation coefficient. Weighted Pearson correlation was used to analyze the spatial correlations over the census tracts, considering the number of patients treated in each tract as weights. For nonparametric data, such as the monthly confirmed dengue cases, Spearman’s rank correlation coefficient was employed. The cross-correlations were calculated using programs developed in our research group, considering a lag and an advance up to six months. Given the seasonality of the phenomena, the cross-correlation results were fitted using a harmonic series truncated to first order, Eq. (1).

1 $$\rho (\tau )=r\,\cos (2\pi (\tau -{\tau }_{0})/T)$$

In Eq. (1), *τ* is the cross-correlation time lag, *τ*_0_ is the time lag value in which the cross-correlation is maximum, *T* is the period of one year, and *r* is the maximum cross-correlation (when *τ* = *τ*_0_). For all tests, *p* values less than 0.05 were considered statistically significant. Statistical analysis was performed with the Statistical Package for the Social Sciences (SPSS) software, the GraphPad Prism program, the Gnumeric Spreadsheet program, and BASH scripts.
